# A New WHO Entity of SMARCB1/INI1-Deficient Undifferentiated Thoracic Tumors: Rare, Aggressive, and Overlooked

**DOI:** 10.7759/cureus.99692

**Published:** 2025-12-20

**Authors:** Chaimae Bekhakh, Anass Haloui, Mohammed Bakhti, Nassira Karich, Amal Bennani

**Affiliations:** 1 Pathology Department, Mohammed VI International University Hospital, Faculty of Medicine and Pharmacy, Mohammed First University, Oujda, MAR

**Keywords:** aggressive thoracic malignancies, rhabdoid morphology, smarcb1-deficient thoracic tumors, swi/snf complex, undifferentiated thoracic neoplasms

## Abstract

SMARCB1/INI1-deficient undifferentiated tumors of the thorax represent a very rare and highly aggressive group of malignancies characterized by rapid progression and considerable diagnostic challenges. Their clinical and radiological features are often nonspecific, while their histological appearance - frequently rhabdoid or undifferentiated - overlaps with several other high-grade thoracic neoplasms. Because these tumors generally lack lineage-defining immunohistochemical markers, diagnosis requires an integrated approach combining morphological assessment, broad immunohistochemical profiling, and confirmation of complete SMARCB1/INI1 loss. In this report, we describe a rare mediastinal case that illustrates the stepwise diagnostic process required to identify this entity. Key aspects of the differential diagnosis are discussed, along with relevant therapeutic implications. Early recognition of such tumors is crucial, as accurate diagnosis can significantly influence treatment strategies and facilitate enrollment in emerging clinical trials targeting SWI/SNF-deficient cancers. Recognition of this recently highlighted yet still unclassified entity within the 2021 WHO classification of thoracic tumors is essential, since timely and precise diagnosis - as demonstrated in our case - directly impacts therapeutic decision-making and enables access to novel targeted therapeutic options.

## Introduction

SMARCB1 (also known as INI1 or SNF5) is a critical tumor suppressor gene located on chromosome 22q11.2. It encodes a core subunit of the SWI/SNF chromatin-remodeling complex, which regulates chromatin accessibility and thereby controls gene expression programs involved in differentiation and proliferation [1]. Loss of SMARCB1 disrupts SWI/SNF function, leading to deregulation of epigenetic programs and contributing to oncogenesis, particularly in tumors exhibiting rhabdoid morphology [2].

Although SMARCB1-deficient tumors are well characterized in pediatric malignant rhabdoid tumors, epithelioid sarcomas, and other soft tissue neoplasms, their occurrence in the thoracic cavity is exceedingly rare [3]. Only a few cases have been reported in the mediastinum, pleura, or lung, often leading to diagnostic confusion due to overlapping features with poorly differentiated carcinomas, sarcomas, or lymphoid neoplasms [3].

Given the rarity and aggressive nature of thoracic SMARCB1/INI1-deficient tumors, reporting additional cases is crucial to enhance awareness, refine diagnostic criteria, and expand therapeutic options. In this article, we present a thoracic SMARCB1/INI1-deficient undifferentiated tumor in an elderly patient, detail the diagnostic process, review differential diagnoses, and discuss molecular mechanisms and therapeutic implications.

## Case presentation

**Figure 1:**
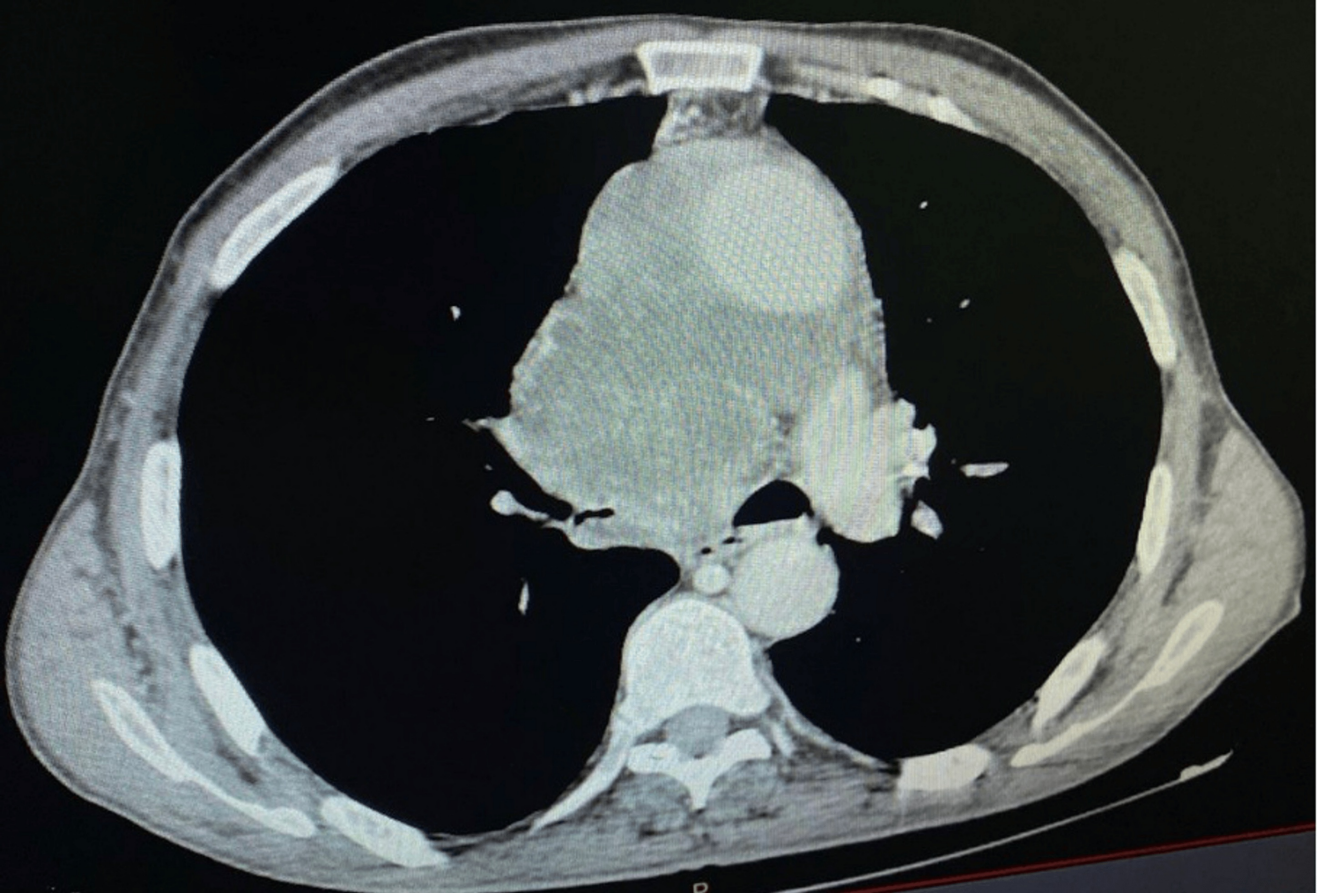
Chest CT scan showing an extensive mediastinal tumor mass with central necrosis and invasion of adjacent structures.

A 71-year-old patient, a chronic smoker with a 40-pack-year history, presented with a one-month history of exertional dyspnea, productive cough with mucopurulent sputum, and dysphonia, in an afebrile context and associated with deterioration of general condition. A chest CT scan revealed a large tumoral mass occupying the upper and middle mediastinum, characterized by irregular contours, poorly defined margins, and a central necrotic component measuring over 10.5 cm. The lesion showed invasive behavior toward adjacent mediastinal, vascular, and tracheobronchial structures, as well as metastatic lymph node involvement (Figure 1).

**Figure 2:**
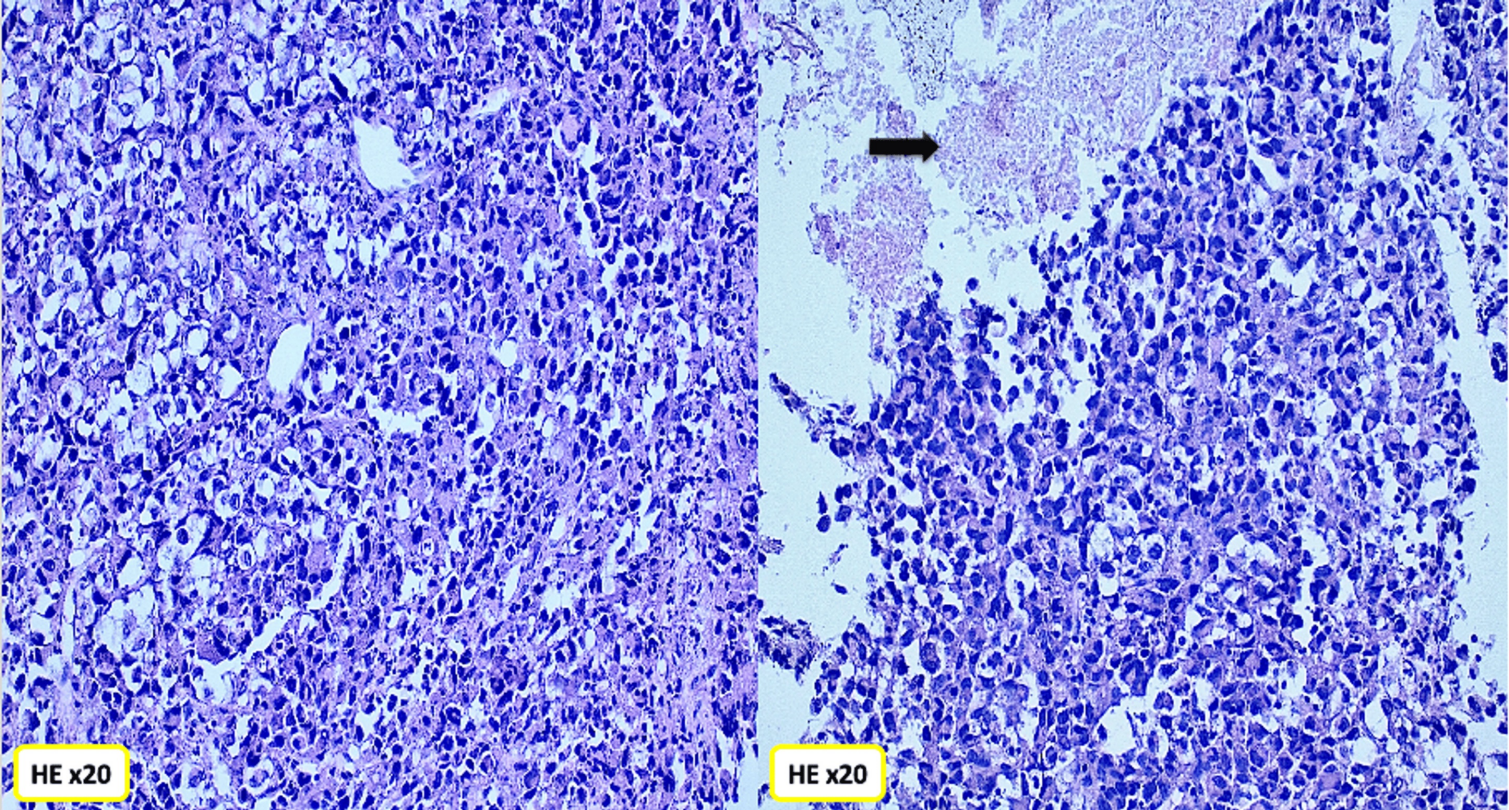
Undifferentiated malignant proliferation composed of clusters of rhabdoid cells with tumor necrosis (black arrow).

**Figure 3:**
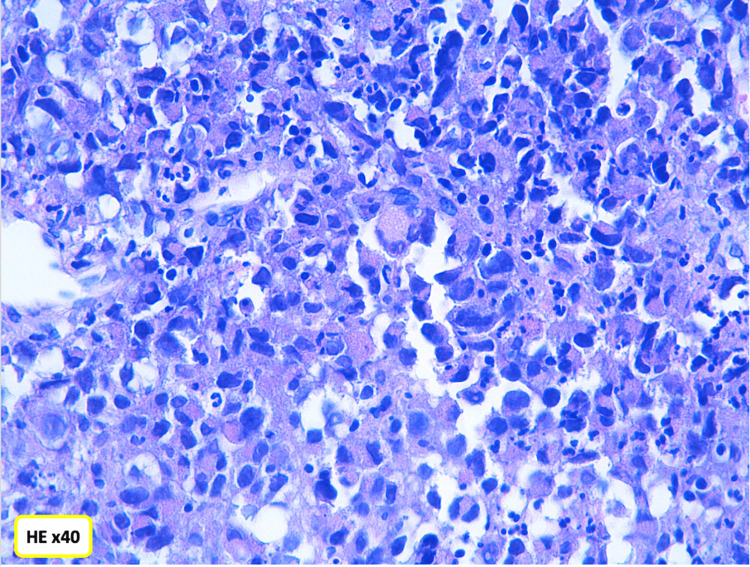
Malignant tumor with a predominance of rhabdoid cells.

A thoracoscopic biopsy of the mediastinal mass was performed. Histopathological examination of the bronchial mucosa revealed a malignant tumor proliferation arranged in sheets and diffuse clusters. The tumor was composed of markedly atypical cells with large, hyperchromatic, and eccentrically located nuclei showing irregular contours and abundant eosinophilic cytoplasm, displaying a rhabdoid morphology. Tumor necrosis was present (Figures 2-3).

**Figure 4:**
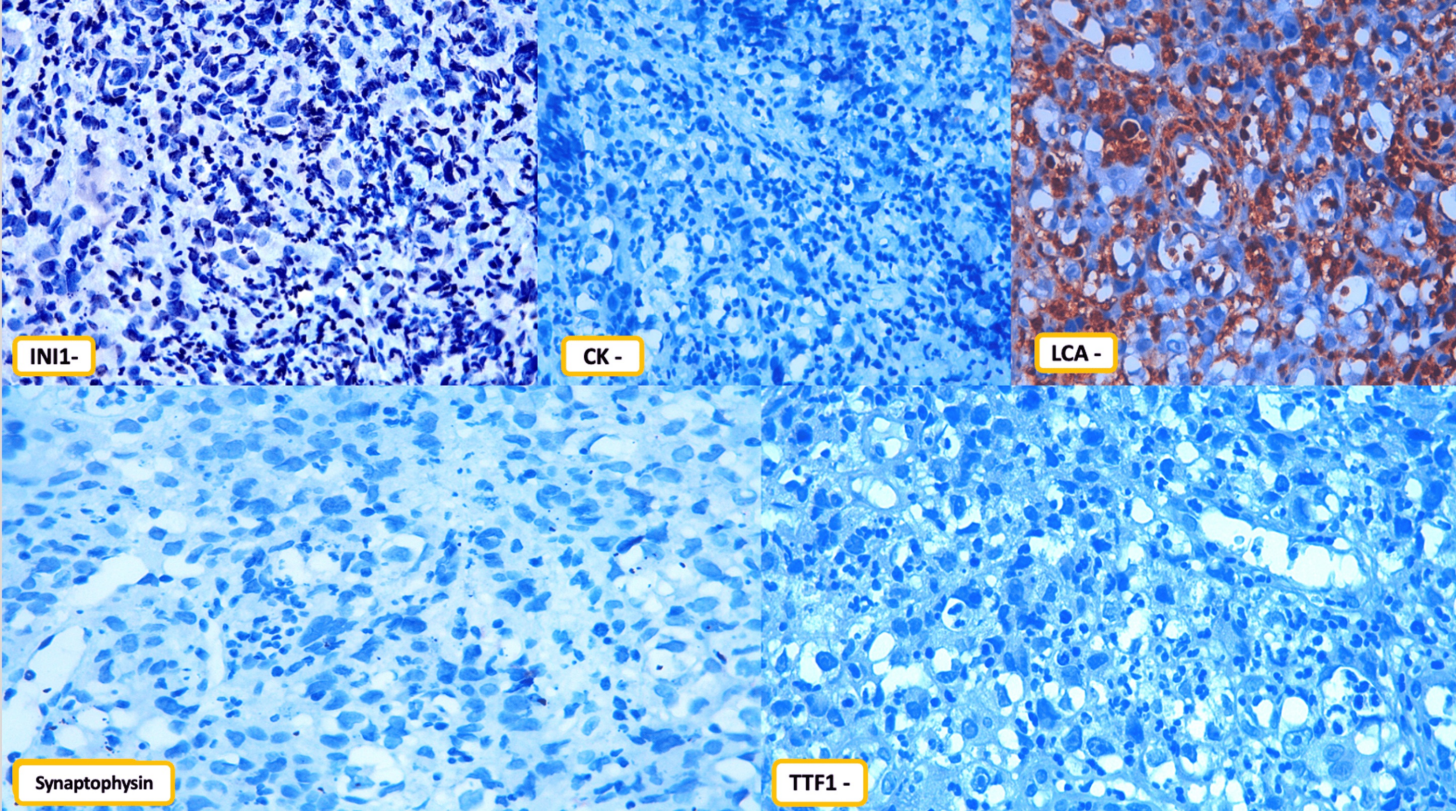
Immunohistochemical analysis demonstrated complete loss of SMARCB1/INI1 expression with an appropriate positive internal control and negative staining for cytokeratin (CK), leukocyte common antigen (LCA), synaptophysin, and thyroid transcription factor-1 (TTF1).

 An immunohistochemical study was performed, revealing the results summarized in Table 1 and illustrated in Figure 4.

**Table 1:** Immunohistochemical profile summary.

Marker	Result	Diagnosis Excluded
INI1 (SMARCB1)	Negative (loss)	Confirms SMARCB1-deficient tumor
Cytokeratin (CK)	Negative	Carcinoma
PSA	Negative	Prostatic carcinoma
PAX8	Negative	Renal/Müllerian origin
TTF1	Negative	Lung adenocarcinoma/thyroid carcinoma
CK5/6	Negative	Squamous cell carcinoma
P40	Negative	Squamous cell carcinoma
EMA	Negative	Epithelial tumors
CD20	Negative	B‑cell lymphoma
CD3	Negative	T‑cell lymphoma
LCA (CD45)	Negative	Hematolymphoid tumor
CD30	Negative	Germ cell tumor
CD117	Negative	Germ cell tumor / thymic carcinoma
PLAP	Negative	Germ cell tumor
CD5	Negative	Thymic carcinoma
TDT	Negative	Lymphoblastic lymphoma
CD138	Negative	Plasmacytoma
CD68	Negative	Histiocytic tumor
Chromogranin	Negative	Neuroendocrine tumor
Synaptophysin	Negative	Neuroendocrine tumor
CD56	Negative	Neuroendocrine tumor
Melan-A	Negative	Melanoma
S100	Negative	Melanoma / nerve sheath tumor
CD31	Negative	Angiosarcoma
CD34	Negative	Angiosarcoma
Desmin	Negative	Rhabdomyosarcoma
AML	Negative	Rhabdomyosarcoma
STAT6	Negative	Solitary fibrous tumor
MDM2	Negative	Liposarcoma
CDK4	Negative	Liposarcoma
Calretinin	Negative	Mesothelioma
WT1	Negative	Mesothelioma
CD99	Weak cytoplasmic	Ewing sarcoma excluded
SOX10	Weak positive	Non-specific

The final pathological report excluded any metastatic origin and concluded that the lesion represented a SMARCB1-deficient undifferentiated tumor (SMARCB1-UT), based on the patient’s smoking history and the distinctive histologic and immunohistochemical features.

Given this definitive diagnosis, the patient was deemed eligible for systemic chemotherapy. The specific chemotherapy regimen will be selected by the treating oncologist, taking into consideration the patient’s overall clinical condition, disease extent (staging), and general health status.

## Discussion

SMARCB1-UT represent a rare and highly aggressive group of malignancies defined by complete loss of INI1/SMARCB1 expression. First recognized in pediatric malignant rhabdoid tumors [4], they have since been increasingly reported in adults across diverse anatomical locations, including the mediastinum, thorax, and retroperitoneum [5]. To obtain an updated overview of thoracic presentations, we performed a PubMed search spanning 2002-2025 using the terms “SMARCB1/INI1-deficient thoracic tumors”, “SMARCB1/INI1-deficient lung carcinoma”, and “SMARCB1/INI1-deficient lung cancer”, identifying 15 cases in the literature (including the present one), as summarized in Table 2.

**Table 2:** Published cases of thoracic SMARCB1/INI1-deficient tumors. Source: Ref. [3,6-10]

Authors	References	Patient	Gender (female/male)	Age (years)	Smoking (Yes/No)	Tumour site	Tumour size (cm)	Lymph node metastasis (Yes/No)	Distant metastasis	Treatment	Follow-up
Yoshida et al., 2018 [6]	6	1	F	33	N	Left pleura	Multiple tumours	N	None	Adjuvant chemotherapy treatment	Died of disease
Haberecker et al., 2022 [7]	7	2	F	20	N	Anterior mediastinum	~4	N	Bone, lung, soft tissue, nerve plexus	Adjuvant chemotherapy treatment	Died of disease
	-	3	M	25	Y	Pleura/thoracic wall, axilla	15.8	Y	Pleura	Adjuvant chemotherapy treatment	Died of disease
	-	4	M	51	Y	Lung, central/hilar	4–5	Y	Pleura	Adjuvant chemotherapy treatment	Died of disease
	-	5	M	56	Y	Pleura	-	N	None	Adjuvant chemotherapy treatment	Died of disease
	-	6	M	76	NA	Lung, subpleural	3.5	N	Not available	Not available	Not available
	-	7	M	73	Y	Lung, central/hilar	5	Y	Liver, adrenal gland	Neoadjuvant / Adjuvant chemotherapy treatment	Died of disease
	-	8	M	64	Y	Pleura	-	N	None	Adjuvant chemotherapy treatment	Died of disease
	-	9	M	72	Y	Lung, central/hilar	9	N	Lung	Adjuvant chemotherapy treatment	Died of disease
	-	10	M	77	N	Posterior mediastinum/esophagus	10	N	None	Neoadjuvant chemotherapy treatment	Died of disease
Zhou et al., 2022 [8]	8	11	M	74	NA	Lung	3.5	N	None	Neoadjuvant radiotherapy	Died of disease
Rickard et al., 2022 [9]	9	12	M	60	N	Lung	8.6	Y	Liver	Neoadjuvant chemotherapy treatment	Alive with disease
Chen et al., 2023 [10]	10	13	M	61	N	Lung	8.8	Y	None	Neoadjuvant / Adjuvant chemotherapy treatment	No evidence of disease
Zagni et al., 2024 [3]	3	14	M	38	N	Lung	5.8	N	None	Neoadjuvant chemotherapy treatment	Died of disease
Present case	-	15	M	71	Y	The upper and middle mediastinum	7	N	None	Adjuvant chemotherapy treatment	Alive with disease

In adults, SMARCB1-UT remains exceedingly uncommon, typically occurring between 20 and 70 years of age [5], with no clear gender predisposition, although some series note a slight male predominance [4]. Risk factors remain poorly understood. Although smoking history is frequently reported in thoracic cases, no clear or consistent association between tobacco exposure and SMARCB1-deficient tumors has been established [3]. Clinically, these tumors present with nonspecific, site-related symptoms such as dyspnea, cough, chest pain, or dysphonia, accompanied in some cases by systemic manifestations, including weight loss and fatigue [3,5]. Radiologically, they characteristically appear as aggressive, infiltrative masses with irregular margins and areas of necrosis, consistent with the findings observed in our patient [5].

Histologically, these tumors display high-grade, undifferentiated morphology composed of sheets of rhabdoid or epithelioid cells with vesicular nuclei, prominent nucleoli, and abundant eosinophilic cytoplasm [3]. Immunohistochemically, loss of INI1/SMARCB1 is a defining feature and is essential for diagnosis [4,5]. Other markers, such as cytokeratins, EMA, or vimentin, may show variable positivity, whereas lineage-specific markers are typically absent. This immunoprofile is critical to exclude differential diagnoses, such as thymic carcinoma, lymphoma, germ cell tumor, and poorly differentiated carcinoma [3] (Table 3). At the molecular level, SMARCB1-UT results from biallelic inactivation of the SMARCB1 gene on chromosome 22q11.23 [11]. SMARCB1 encodes a core component of the SWI/SNF (BAF) chromatin-remodeling complex, which regulates nucleosome positioning and transcriptional accessibility [11]. Its loss leads to profound epigenetic deregulation, driven by an imbalance between SWI/SNF and PRC2 complexes, with EZH2-mediated H3K27 methylation becoming dominant [11]. This creates a repressive chromatin landscape conducive to malignant transformation. SMARCB1 loss also enhances MYC-driven transcriptional programs and proliferative signaling pathways [12]. Notably, these tumors frequently exhibit a low mutational burden, suggesting that SMARCB1 loss alone may be sufficient to initiate tumorigenesis [13]. These molecular insights support the rationale for EZH2 inhibitors, which aim to counteract PRC2-mediated repression [14].

**Table 3:** Differential diagnoses of SMARCB1-deficient undifferentiated tumors in adult mediastinal masses. Table constructed based on information derived from Zagni et al. [3] and Song et al. [15], both published under Creative Commons license.

Differential Diagnosis	INI1 Status	Key Immunohistochemical Markers	Distinguishing Features/Clues
Thymic carcinoma	Retained	CD5+, CD117+, p40+	Anterior mediastinal origin, keratin-positive, NUT-negative
Primary mediastinal large B-cell lymphoma (PMBCL)	Retained	CD45+, CD20+, PAX5+, CD30+	Bulky mediastinal mass, keratin-negative
Mediastinal germ cell tumors	Retained	SALL4+, OCT3/4+, CD30+	Young adult males, high AFP/β-HCG
Undifferentiated lung carcinoma	Retained	TTF-1+, Napsin A+, CK7+	Lung origin on imaging
Ewing/CIC sarcoma	Retained	CD99+, NKX2.2+/WT1 C-terminal+	Small round blue cell tumor, gene fusions
Epithelioid MPNST	Lost	S100+ focal, SOX10+ focal	Peripheral nerve association ; INI1 loss
SMARCA4-deficient thoracic tumor	Retained	SMARCA4 loss, SMARCA2 loss	Heavy smoker; rhabdoid morphology
NUT carcinoma	Retained	NUT+ nuclear staining	Aggressive midline carcinoma
Synovial sarcoma	Retained	TLE1+, focal CK+, STAT6+	SS18-SSX fusion
Extrarenal malignant rhabdoid tumor	Lost	CK+, vimentin+, EMA+	Mostly pediatric

Therapeutically, SMARCB1-UT remain difficult to manage due to their aggressive clinical course and limited response to conventional chemotherapy [5,14]. When achievable, surgical resection represents the preferred initial treatment, often followed by multimodal therapy, including radiotherapy and systemic treatment [5]. Emerging strategies targeting epigenetic dysregulation - particularly EZH2 inhibitors and other chromatin-modifying agents - are currently under investigation and may offer promising future therapeutic avenues for this rare and challenging entity [14].

## Conclusions

SMARCB1-UT represent a rare and aggressive entity characterized by distinctive histopathological, immunohistochemical, and molecular features. This case highlights the diagnostic challenges posed by their nonspecific clinical presentation and broad differential diagnosis, particularly in older patients with a history of smoking. Loss of INI1 expression remains the cornerstone for confirming the diagnosis. A thorough evaluation integrating imaging, morphology, immunohistochemistry, and genomic data is essential to avoid diagnostic pitfalls, especially with mimickers such as poorly differentiated carcinomas, lymphomas, sarcomas, and thymic neoplasms.

Although therapeutic strategies remain limited and not yet standardized, early recognition is crucial for guiding appropriate management and improving outcomes. Growing insights into SMARCB1 biology and epigenetic vulnerability offer promising avenues for targeted therapies, underscoring the importance of reporting additional cases to refine our understanding of this emerging tumor entity.
